# Isolated high altitude psychosis, delirium at high altitude, and high altitude cerebral edema: are these diagnoses valid?

**DOI:** 10.3389/fpsyt.2023.1221047

**Published:** 2023-08-04

**Authors:** Katharina Hüfner, Marika Falla, Hermann Brugger, Hannes Gatterer, Giacomo Strapazzon, Iztok Tomazin, Ken Zafren, Barbara Sperner-Unterweger, Paolo Fusar-Poli

**Affiliations:** ^1^Division of Psychiatry II, Department of Psychiatry, Psychotherapy, Psychosomatics and Medical Psychology, Medical University of Innsbruck, Innsbruck, Austria; ^2^Institute of Mountain Emergency Medicine, Eurac Research, Bolzano, Italy; ^3^Department of Neurology/Stroke Unit, Hospital of Bolzano (SABES-ASDAA), Bolzano, Italy; ^4^Department of Anesthesia and Intensive Care Medicine, Medical University of Innsbruck, Innsbruck, Austria; ^5^Department of Family Medicine, Faculty of Medicine, University of Ljubljana, Ljubljana, Slovenia; ^6^Mountain Rescue Association of Slovenia, Kranj, Slovenia; ^7^Department of Emergency Medicine, Stanford University Medical Center, Palo Alto, CA, United States; ^8^Department of Emergency Medicine, Alaska Native Medical Center, Anchorage, AK, United States; ^9^Department of Psychosis Studies, Institute of Psychiatry, Psychology and Neuroscience, King's College London, London, United Kingdom; ^10^Department of Brain and Behavioral Sciences, University of Pavia, Pavia, Italy

**Keywords:** acute mountain sickness, altitude, high altitude cerebral edema, psychosis, delirium

## Abstract

Psychosis is a psychopathological syndrome that can be triggered or caused by exposure to high altitude (HA). Psychosis can occur alone as isolated HA psychosis or can be associated with other mental and often also somatic symptoms as a feature of delirium. Psychosis can also occur as a symptom of high altitude cerebral edema (HACE), a life-threatening condition. It is unclear how psychotic symptoms at HA should be classified into existing diagnostic categories of the most widely used classification systems of mental disorders, including the Diagnostic and Statistical Manual of Mental Disorders (DSM-V) and the International Statistical Classification of Diseases and Related Health Problems (ICD-11). We provide a diagnostic framework for classifying symptoms using the existing diagnostic categories: psychotic condition due to a general medical condition, brief psychotic disorder, delirium, and HACE. We also discuss the potential classification of isolated HA psychosis into those categories. A valid and reproducible classification of symptoms is essential for communication among professionals, ensuring that patients receive optimal treatment, planning further trips to HA for individuals who have experienced psychosis at HA, and advancing research in the field.

## 1. Introduction

**Figure 1:**
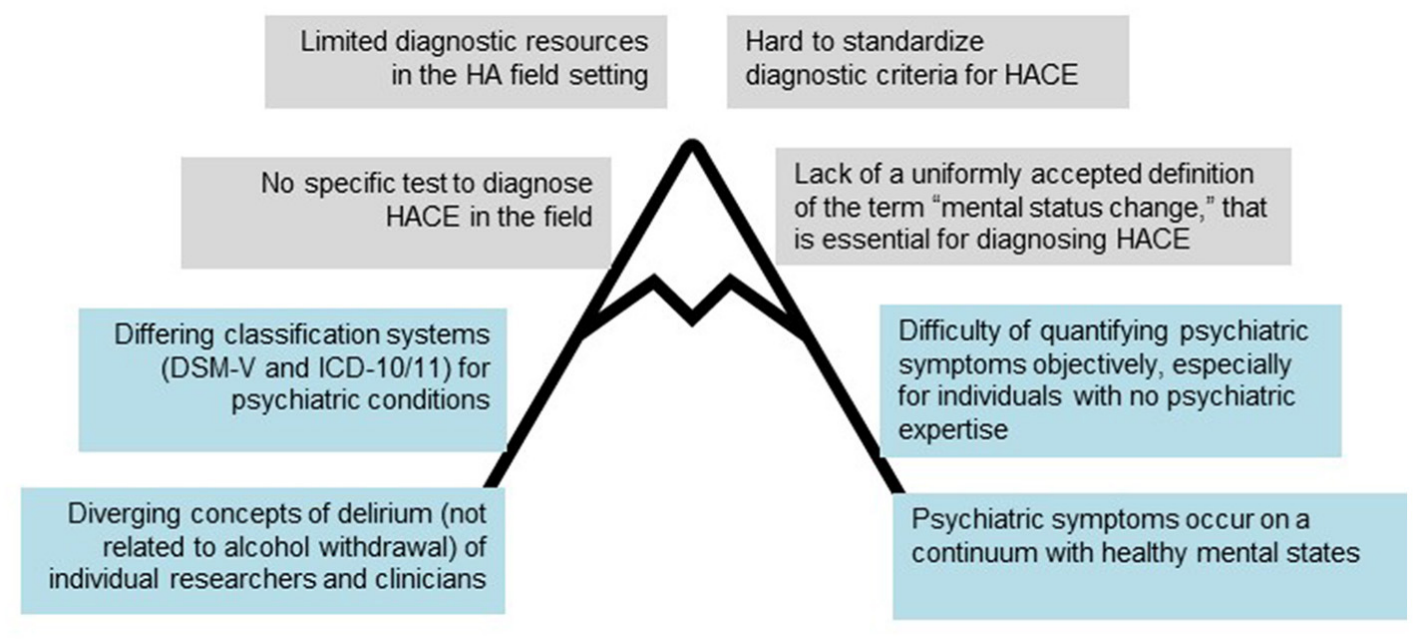
Diagnostic difficulties encountered in trying to identify and categorize mental symptoms at high altitude (HA). Barriers specific to HA are in gray. Barriers also encountered in other settings are in blue.

Medical professionals and mountaineers are aware of somatic complications of high altitude (HA) exposure, but research, clinical guidelines, and knowledge among mountaineers concerning mental symptoms at HA are limited. Psychopathological changes, such as altered consciousness or attention, hallucinations, and delusions can occur at HA (1–3). These changes have most commonly been linked not only to underlying organic processes triggered by hypoxia but also to infections, environmental conditions, or drugs. Psychogenic factors such as social isolation or mental stress may also play a role. Compared to somatic symptoms, mental symptoms at HA are underreported (4). However, it is critical to provide appropriate treatment and prognostic recommendations, to recognize mental symptoms as accurately as somatic symptoms, and to classify them correctly using current diagnostic categories. In our opinion, several issues, some of which are universal and others of which are specifically related to HA, hinder this process (Figure 1). In this hypothesis article, we provide guidance on how to classify psychotic symptoms correctly using standardized diagnostic criteria.

## 2. Psychosis as a psychopathological syndrome at high altitude

A syndrome is a collection of individual symptoms that commonly occur together. A syndrome with a specific cause is called a disease. According to the dimensional assessment of the Diagnostic and Statistical Manual of Mental Disorders, Fifth Edition (DSM-V) (5), psychosis is a psychopathological syndrome characterized by symptoms of hallucinations, delusions, disorganized thought/speech, abnormal psychomotor behavior, and negative symptoms, as well as impaired cognition, depression, and mania. At least two of the symptoms must be present for the diagnosis of psychosis, with at least one of the symptoms being hallucinations, delusions, or disorganized thought/speech. The definition is comparable to ICD-11 although the DSM does not explicitly list impaired cognition, depression, and mania (6). Hallucinations and delusions are generally considered to be the defining and most prevalent features of psychosis (7). Symptoms of psychosis and other mental symptoms occur on a continuum with normal mental states and are not binary entities. Psychotic symptoms can occur in the general population, for example, the personality trait of schizotypy is associated with being prone to psychotic symptoms (8, 9). Stressful life events or mental trauma are potential risk factors for psychotic symptoms in the general population (10). Physiological disturbances such as sleep deprivation or sensory deprivation can trigger psychotic symptoms in healthy individuals (11, 12).

Psychotic symptoms at HA have mostly been described in the lay literature, case reports, and small case series.

“*I climbed to the summit in 4 days as a member (and doctor) of a small team of Slovenian climbers. I had almost no acclimatization but a lot of motivation… Suddenly the hallucinated “mountain guides” started to talk to me with very sweet and energetic advice: “Jump down the east face and in a few seconds you will be on a flat, safe place 2,000 m lower. This will solve all your problems.” So I was standing there, at the very edge of the east face, prepared to jump because these voices almost convinced me that jumping down the face was the best or only solution to my problems. I almost jumped and this would have meant death with a 100% certainty… With coming lower, hallucinations slowly disappeared. I reached our bivouac at 6,500 m after several hours of climbing, without hallucinations but still with cognitive impairment.”—Iztok Tomazin, Dhaulagiri winter alpine-style ascent in December 1987; slightly adapted* *(**3**)*.

An analysis of the lay literature suggests that hallucination is a very prominent feature of psychosis at HA and was present in 83% of the analyzed episodes (3). The so-called third man/person syndrome is the felt presence of another person who can often also be seen or heard. This was the most common hallucination, occurring in 54% of cases of psychosis at HA (3). The third man/person syndrome has mostly been reported by individuals exposed to high levels of stress, often in extreme environments. Many accounts have been collected in a book, *The Third Man Factor*, by John Geiger (13) and also continue to appear in the media. For example, the climber Elisabeth Revol forced to bivouac at HA on Nanga Parbat in winter, a very difficult situation, described an interaction with a woman who offered her hot tea to protect her from the freezing cold in return for a shoe. She suffered severe frostbite. Visual, acoustic, or somesthetic hallucinations can also occur at HA.

**Figure 2:**
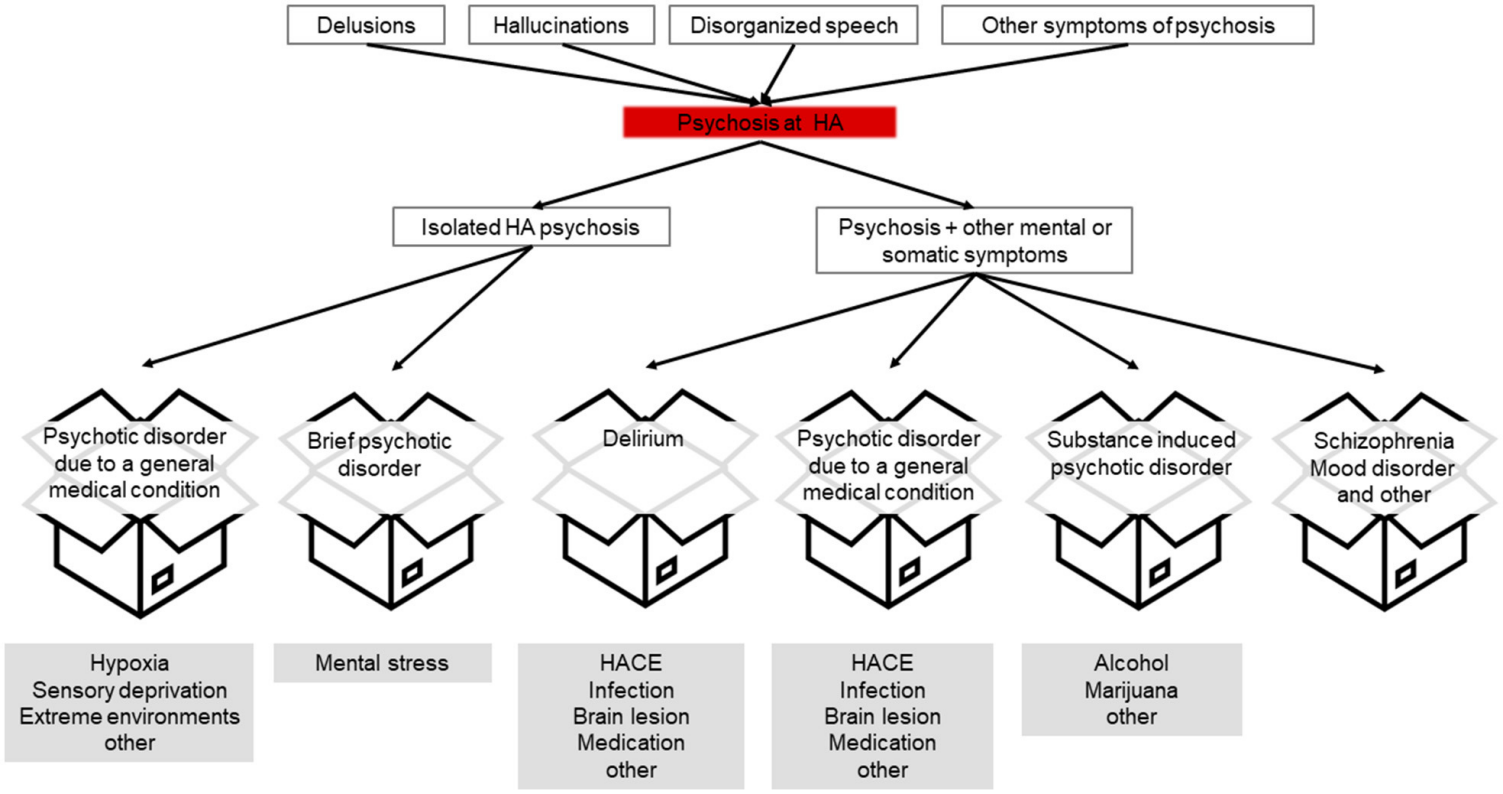
Pathway from psychotic symptoms to possible diagnostic categories (boxes). Possible underlying factors or conditions at high altitude are indicated in gray.

Psychotic symptoms are transdiagnostic, meaning that they are not the characteristics of a single disorder but can be found in various conditions. Psychosis at HA can occur in the absence of other clinical features such as isolated HA psychosis or be accompanied by somatic or mental symptoms (Figure 2). Psychotic symptoms together with other mental symptoms commonly occur in mental disorders such as schizophrenia and mood disorders, Lewy body dementia, delirium, or substance abuse (14). Foreign travel can trigger psychosis through stressors such as environmental conditions, unaccustomed physical exertion, and psychosocial factors, all of which can also occur in HA travel (15).

## 3. Can diagnostic categories already present in DSM V be useful to assign psychosis at HA to a diagnostic category?

We also refer to ICD-11 in case substantial differences to DSM-V exist.

### 3.1. Psychotic disorder due to a general medical condition

Symptoms of psychosis, such as hallucinations or delusions, can occur as a consequence of a medical condition. They are then classified as psychotic disorders due to a general medical condition in the DSM-V (5) (Table 1) and called secondary psychotic syndromes in ICD-11 (World Health Organization, 2019/2021). At sea level and low altitude, epilepsy is recognized as the most common underlying factor of psychotic disorder due to a general medical condition (16). At high altitude, HACE can be the underlying condition. HACE is a life-threatening cause of psychosis that requires emergent treatment. Psychosis might be an important early warning sign of HACE. However, this needs further validation in clinical studies.

**Table 1:** Diagnostic criteria of “psychotic disorder due to a general medical condition” based on the DSM-V (5).

A. Prominent hallucinations or delusions.
B. There is evidence from the history, physical examination, or laboratory findings that the disturbance is the direct physiological consequence of a general medical condition.
C. The disturbance is not better accounted for by another mental disorder.
D. The disturbance does not occur exclusively during the course of delirium.
E. The disturbance causes clinically significant distress or impairment in social, occupational, or other important areas of functioning.

The category of psychotic disorder due to a general medical condition may also apply to isolated HA psychosis, a syndrome that is often characterized by prominent hallucinations (Table 1, criterion A). The evidence is unclear whether isolated HA psychosis is a direct physiological consequence of hypoxia (Table 1, criterion B). This is likely the case because symptoms of isolated HA psychosis disappear without sequelae once an individual reaches a lower altitude (3, 17). It is debatable whether hypoxia caused by ascent to HA can be considered to be a general medical condition although this would account for the generally benign nature and reversibility of symptoms once the causative condition is removed.

### 3.2. Brief psychotic disorder

The category of brief psychotic disorder in the DSM-V (similar to an “acute and transient psychotic disorder” in ICD-11) requires a duration of at least 1 day and is often associated with a stressful life event (Table 2). Travel to HA could be considered a stressful life event. Stress and exhaustion have been associated with psychotic symptoms in high-performance endurance athletes (18, 19). Symptom duration in isolated HA psychosis can be shorter than 24 h (Table 2, criterion B). It is unclear whether isolated HA psychosis carries a risk of recurrence or predisposes an individual to other psychotic disorders as does brief psychotic disorder (20). Based on media reports, individuals who have experienced psychosis at HA return to and remain at their premorbid level of functioning once they descend to lower altitudes (Table 2, criterion B). While brief psychotic disorders often require treatment with antipsychotics, this has not been reported in isolated HA psychoses.

**Table 2:** Diagnostic criteria of brief psychotic disorder according to the DSM-V (5).

A. Presence of one or more of the following symptoms. At least one of these must be (1), (2), or (3): 1. Delusions. 2. Hallucinations. 3. Disorganized speech (e.g., frequent derailment or incoherence). 4. Grossly disorganized or catatonic behavior.
B. Duration of an episode of the disturbance is at least 1 day but less than 1 month, with eventual full return to premorbid level of functioning.
C. The disturbance is not better explained by major depressive or bipolar disorder with psychotic features or another psychotic disorder, such as schizophrenia or catatonia, and is not attributable to the physiological effects of a substance (e.g., a drug of abuse, a medication), or another medical condition.

### 3.3. Delirium

When psychotic symptoms occur in association with disturbances in attention and awareness caused by external triggers, this constitutes organic brain dysfunction (classified as “delirium” in the DSM-V and ICD-11; Table 3) (5, 6, 22). Delirium at HA can occur in the context of HACE or as a result of systemic conditions such as infection or dehydration (23–26). The diagnostic criteria of “delirium” are comparable between ICD-11 and the DSM-V (27).

**Table 3:** DSM-V TR diagnostic criteria for delirium (21)^*^.

A. Disturbance in attention (i.e., reduced ability to direct, focus, sustain, and shift attention) accompanied by reduced awareness of the environment.
B. The disturbance develops over a short period of time, usually hours to a few days, represents an acute change from baseline attention and awareness, and tends to fluctuate in severity during the course of a day.
C. An additional disturbance in cognition, including memory deficit, disorientation, language, visuospatial ability, or perception.
D. The disturbances in A and C are not better explained by a pre-existing, established, or evolving neurocognitive disorder and do not occur in the context of a severely reduced level of arousal such as coma.
E. There is evidence from the history, physical examination, or laboratory findings that the disturbance is a direct physiological consequence of another medical condition, substance intoxication or withdrawal (i.e., caused by a drug of abuse or a medication) or exposure to a toxin or is due to multiple etiologies.

^*^Diagnostic criterion A has been clarified in the text revision (TR) version of DSM-V.

In a field study of mostly well-acclimatized individuals at the Everest Base Camp, there was only one case of delirium (28). This is a low incidence compared to previous literature (3, 24). When a diagnosis of delirium is made, it is essential to attempt to identify the underlying pathology (Table 3, criterion E). At HA, it is critical to consider HACE as a possible cause. HACE is a life-threatening condition that must be treated emergently by descent. Isolated HA psychosis should not be classified as delirium because the criteria of delirium, such as changes in attention and awareness or arousal (Table 3, criterion A, D), are not a characterizing feature of isolated HA psychosis. There are several good assessment tools available for the diagnosis of delirium. The Confusion Assessment Method (CAM) must be used with a standardized interview by a trained interviewer (29). The Delirium Observation Screening Scale (DOSS) (30) is a screening tool that only requires observation of patients with no specific cognitive testing.

## 4. High altitude cerebral edema

While most clinicians working at HA have a personal gestalt of the symptoms of HACE, there are no uniform diagnostic criteria for the psychopathology defining HACE. HACE is distinguished from AMS by disturbances in consciousness that may progress to coma, psychiatric changes, confusion, and ataxia (31). The term “altered mental status,” often used to describe the mental symptoms in HACE, is a descriptive term that includes a range of psychopathological symptoms. In the original consensus report, mental status was defined by the following categories: no change in mental status, lethargy/lassitude, disoriented/confused, stupor/semiconsciousness, and coma (32). In some settings, mental status change is equivalent to delirium with disturbances in attention, awareness, cognition, and level of consciousness (33) (Table 3). For a diagnosis of HACE, the STAR data reporting guidelines defined change in mental status as disturbances of orientation (to person, place, and time) or somnolence/confusion/coma (34) (Table 4). Other guidelines require drowsiness, confusion, or irritability (35). In a study focused primarily on ataxia in HACE, mental status changes consisted of disturbances in consciousness (79%), lassitude (41%), apathy (36 %), drowsiness (33%), psychological changes (27%), disorientation (14%), and hallucinations (3%) (36). Psychological changes comprised strange or irrational behavior, emotional changes, and impaired long-term or short-term memory. The psychopathological symptoms of HACE are multifaceted. There are no standardized assessment tools to assess symptoms. While coma is relatively easy to diagnose, judgments regarding lethargy or lassitude differ among evaluators. We believe that rating the changes in consciousness using the Glasgow Coma Scale or other validated scales to assess consciousness such as the AVPU scale (alert, responsive to voice, responsive to pain, or unresponsive) can be easily administered by first responders and others in a pre-hospital setting without formal training (37), would be a step forward to solving this problem. Using an assessment tool for delirium could also aid in diagnosis.

**Table 4:** Diagnostic criteria of HACE (40).

A) Symptoms of AMS or HAPE	A) No Symptoms of AMS or HAPE
B) Mental status change AND/OR ataxia	B) Mental status change AND ataxia
C) Onset within 24–72 h following a gain in altitude	

Mental status change refers to disturbances of orientation (to person, place, and time) or altered consciousness with confusion, somnolence, or coma (34). Ataxia refers to an unsteady gait or inability to walk in a straight line heel-to-toe (tandem gait). AMS, acute mountain sickness; HACE, high altitude cerebral edema; HAPE, high altitude pulmonary edema.

The evaluation of possible HACE should include a full mental status examination and not be restricted to consciousness. Only then would it be possible to make a full assessment of psychopathological status, which is necessary to diagnose psychiatric conditions at any altitude. Manic or depressive symptoms as well as anxiety can be triggered by HA. It is unclear whether these symptoms also occur in HACE (2, 3, 24, 38). A pre-existing psychiatric condition is a risk factor for developing AMS. It is unclear whether this is also the case for HACE (39).

## 5. Discussion and conclusion

The diagnosis of psychotic symptoms at HA is essential for communication among professionals for ensuring patients receive optimal treatment, for planning further trips to HA for individuals who have experienced psychosis at HA, and for advancing research in the field. If clinical and research databases of such relatively rare, but potentially dangerous, conditions are developed, it will be essential to reproducibly classify symptoms into diagnostic categories. There are additional factors, including legal and insurance considerations, that make it important to assign psychotic symptoms to the correct diagnostic categories. For people going to HA, past medical history, including psychiatric and especially psychotic episodes, is important information for guides, leaders, and other assessors of risk. Different diagnostic categories carry different levels of concern. The most dangerous cause of delirium at HA is HACE, a life-threatening condition that requires emergent treatment. Specific diagnostic criteria defining the psychopathology in HACE might help differentiate HACE from other HA-associated mental disorders. There is no current diagnostic category that is a perfect fit for isolated HA psychosis. Isolated HA psychosis is attributed primarily to hypoxia and is usually benign and self-limiting, resolving after descent and restoration of normoxia. Isolated HA psychosis exacts a personal cost to the individual who lived through the experience, as well as having prognostic and legal implications. Attribution to HA and complete recovery can be reassuring in the long term and can help to reduce stigma. Further research and clinical case descriptions are needed to evaluate the best diagnostic category for isolated HA psychosis. It is also possible that the existing diagnostic categories do not adequately describe isolated HA psychosis. Available evidence suggests the following distinct features of isolated HA psychosis: (a) It occurs in the context of a recent gain in altitude in an individual without prior history of psychosis at sea level. (b) It occurs without additional psychopathology such as an altered level of consciousness or prominent changes in attention and awareness (suggesting for example HACE or delirium). (c) The affected individual is able to descend by walking without physical assistance (unless physical injuries are present). (d) The symptoms disappear without sequelae once the individual reaches a lower altitude. These criteria will require testing and validation in clinical practice.

## Data availability statement

The original contributions presented in the study are included in the article/supplementary material, further inquiries can be directed to the corresponding author.

## Author contributions

Initial project conceptualization and drafting of initial manuscript: KH. Development of presented hypothesis: KH, HB, BS-U, and PF-P. Discussion and further evolution of presented hypothesis, important revisions of manuscript, and approval of final version: all authors.
